# Impact of relational leadership on employees’ unethical pro-organizational behavior: A survey based on tourism companies in four countries

**DOI:** 10.1371/journal.pone.0225706

**Published:** 2019-12-09

**Authors:** Xianchun Zhang, Zhu Yao

**Affiliations:** 1 ASEAN Tourism Research Center of China Tourism Academy, Guilin Tourism University, Guilin, Guangxi, China; 2 Sino-Indonesia Tourism Academy, Trisakti School of Tourism, Jakarta, Indonesia; 3 School of Economics and Management, Tongji University, Shanghai, China; Shandong University of Science and Technology, CHINA

## Abstract

Based on the theory of social construction and self-consistency, this study aims to investigate the mechanism of relational leadership’s role in employees’ unethical pro-organizational behavior (UPB) from the perspective of moral identity and ethical climate. We found that relational leadership negatively correlates with the instrumental ethical climate, positively correlates with caring ethical climate, and exerts no significant impact on the rule ethical climate. Instrumental ethical climate and caring ethical climate mediate the relationship between relational leadership and employees’ unethical pro-organizational behavior. In addition, moral identity negatively moderates the relationship between instrumental ethical climate and employees’ unethical pro-organizational behavior, and between caring ethical climate and employees’ unethical pro-organizational behavior. Furthermore, moral identity positively moderates the relationship between a rule ethical climate and employees’ unethical, pro-organizational behavior.

## Introduction

Recent years have witnessed an upsurge in corporate-ethics incidents such as Thai tour guides forcing tourists to shop, and Indonesian tour guides insulting tourists. These incidents not only resulted in serious losses to the enterprises and their stakeholders, but exerted a significant negative impact on the market environment and the healthy development of the broader economy and society. Such unethical behavior often closely correlates with the unethical behavior of employees [1]. To date, most studies on unethical behavior have suggested that employees display unethical behavior for personal gain, for revenge, or to harm colleagues [2]. However, in actual work, employees can also exhibit unethical behavior to sustain or increase the interests of their organization—acts such as tampering with financial data to increase the value of the organization’s stock, or concealing negative information from the public. Umphress et al. characterized this behavior as unethical pro-organizational behavior (UPB) [3]. To protect their interests, organizations are generally able to either ignore or acquiesce, as unethical behavior in the long run would damage the organization’s image and interests. If to the organization’s benefit employees of tourism enterprises deliberately hide crucial information from customers, profits may increase in the short term. However, in the long term customers may well feel “cheated,” thereby refusing to purchase the company’s products or services later on. There is thus a need to raise awareness amongst organizational leaders of the hazards of such behavior, and to identify ways of reducing employee UPB. This would be of great significance in helping organizations achieve healthy and lasting benefits [3,4].

Most prior studies considered UPB to be a product of positive relationship exchange and organizational identity, and that organizational commitment and organizational identity affect UPB [5,6]. Recently, some experts have attributed the occurrence of UPB to the positive relationships that employees enjoy with some forms of leadership, such as transformational or ethical leadership [7,8,9], and are committed to building the relationship-oriented leadership of organizations with free communication and mutual trust among members. Research on the impact of leadership on employees’ UPB remains rarely involved—and most studies have been conducted in Western countries [7,8]. On the one hand, research focusing on the ASEAN cultural milieu remains highly limited; and in recent years the number of ethical incidents among ASEAN tourism enterprises has increased manifold, all of which highlights the necessity of further investigating this phenomenon. On the other hand, owing to the impact of Chinese culture, southeast Asia attaches great importance to human relations. Accordingly, it is necessary to study relational leadership. However, the impact of relational leadership on employee UPB and the mechanism of action remain unclear. Current research has not delivered direct results [10,11,12].

Hence, taking the employees of ASEAN tourism enterprises as study object, this paper aims to explore the mechanism of relationship among leadership, ethical climate, and UPB, and to investigate the regulatory effect of moral identity. Of note, we make the following theoretical contributions: (i) the relationship between relational leadership and UPB is discussed, and the effect of relational leadership on UPB is revealed. This may be most helpful in encouraging scholars to explore UPB from a new perspective of leadership style, and in advancing leaders’ understanding of the harm that UPB might bring to the organization; (ii) based on the theory of social construction, the study identifies the mediating role of ethical climate between relational leadership and UPB; this supplements the path between relational leadership and UPB, and provides new ideas for scholars who conduct follow-up studies; (iii) this study assesses the moderating effects of moral identity, which helps to expand the boundary conditions between ethical climate and the UPB, in turn enabling managers to better inhibit the UPB of employees. Finally, in our conclusions we provide theoretical and empirical support to those who wish to avoid or reduce the UPB of organizational members.

## Theory and hypothesis

### 2.1 Relational leadership and ethical climate

Relational leadership denotes a leader’s leadership style in implementing the development prospects of an organization through a series of measures (including care, tolerance, authorization, and fairness to subordinates). In addition, relational leadership is people-centered, and strives to create a harmonious atmosphere among employees. Typically, this is achieved primarily through an emphasis on the maintenance of harmonious interpersonal relationships with subordinates; the creation of a caring and human-oriented organizational environment; and by providing subordinates a measure of independent development space [13]. In any case, relational leadership places leadership behavior in various complex relationships within organizations. The key issues here are: how to achieve effective communication with organization members; how to create good organizational relationships; and how to augment organizational member formation and alignment.

The five dimensions of relational leadership are as follows: care; empowerment/authorization; fairness; tolerance/inclusiveness; and vision [14]. Care inheres in the level of concern shown by a leader for the career development of the employee. Empowerment/authorization denotes the degree of autonomy that an employee enjoys at work. Fairness refers to employees’ subjective feelings about job-assignment and salary equity in terms of job assignment and salary. Tolerance/inclusiveness implies the degree of acceptance of recommendations made by a leader. Lastly, vision denotes the quality of leaders’ plans for the employees of development prospects. Currently, studies on relational leadership are limited. Akram et al. report that relational leaders in relationship-oriented enterprises give subordinates an increased sense of belonging; actively do their work [15]; and contribute to the enterprise. In addition, some experts demonstrate that the higher the level of leadership concern for the employee, the better the relationship between the two. Moreover, increased concern also enhances communication and openness between leader and employee; decreases conflict among employees; helps employees contribute towards organizational aims; and increases the ability of the organization to design and assess sustainable business models [16]. In addition, relational leadership may also increase daily communication among employees; enhance their level of mutual trust; and create a psychologically secure environment of trust within the team [17]. Relational leadership is ethically responsible to employees by sustaining good relationships with employees. Furthermore, such leadership helps to further sensitize leaders to the daily affairs of employees, and highlights the existence of leaders’ moral responsibility [18].

UPB refers to those who, in motivating an organization or its members to quickly achieve work goals, violate the core values of society, morality, law, and/or reasonable behavioral standards [3]. Notably, behavior that benefits an organization might also benefit the actor. Thus, UPB does not deviate from self-interested unethical behavior, and its motivation is often a combination of altruism and self-interest [6]. Lately, UPB has become a leading subject for academic discussion. Tian et al. revealed that power distance positively regulates the relationship between pressures that encourage unethical behavior and unethical earnings management behavior [19]. Scholars have also reported a negative relationship between UPB and mindfulness [20]. Undoubtedly, recent research has pushed leadership factors to the foreground: some studies have demonstrated that transformational leadership increases employees’ unethical behavior [7]; and one paper has reported an inverted U-shaped relationship between ethical leadership and employee UPB.

Based on social construction theory, employees form consciousness and cognition as a function of differing environments or leadership styles—out of which they subsequently engage in corresponding behavior. Relational leadership creates a flexible and relaxed work environment that leads to the development of employees’ autonomy and job satisfaction [21]. However, it may also result in excessive autonomy, or an overly relaxed attitude amongst employees; these in turn can generate overconfidence or narcissism [22], tending to create UPB that may benefit the organization or colleagues, but that is exposed to moral hazard [23]. Existing literature shows that relational leadership is readily influenced by several uncertain factors in decision-making, which may further worsen the external environment of enterprises, and lead to unethical behavior within the organization [10,24]. Moreover, in providing care and fostering independence, the trust relationship between leaders and employees is enhanced [25], thus stimulating a reciprocal sense of motivation amongst employees [26]. And significantly, reciprocity is a major cause of individual UPB [8,9].

Based on the arguments presented above, this study proposes the following hypothesis:

*Hypothesis 1*: *Relational leadership positively correlates with the employee of UPB*.

### 2.2 The mediation effect of ethical climate

Ethical climate denotes a common cognition formed by members of an organization in terms of the parameters of ethical behavior, and of how to deal with ethical issues [27]. Having been verified and supported by several scholars, three types of ethical atmosphere are now commonly accepted: instrumental; caring; and rule [28,29]. An Instrumental ethical climate (IEC) is one in which employees’ behavioral decisions are made largely to satisfy their interests, with little or no regard for the impact of their actions upon others. In a Caring ethical climate (CEC), employees fully consider the impact that behavioral decisions might have on others, and are concerned for the overall interests of others and for the organization. A Rule ethical climate (REC) signifies that employees must abide by organizational rules—a value orientation that conforms to the group norms [29,30,31]. Relational leadership consists in the fact that leaders should treat employees as “humanism,” attempt to understand them, and strive to create an atmosphere of support and trust. Under the IEC or self-interest climate, employees may employ unscrupulous or unethical means to maximize individual or organizational interest. However, if leaders demonstrate great care, and help their employees, they may not only enable employees to gain support and respect, but also enhance their work commitment, contribute to the organization and society to reward leaders, and decrease employees’ self-interested behavior [31]. Perhaps, leaders’ concern and support for employees could provoke individuals’ high sense of identity, emotional commitment, and altruism to the organization, which readily forms a friendly and caring ethical climate. Relational leadership is not only directed towards the interests of the organization, but also actively empowers employees, giving them more freedom and flexibility at work. When employees face ethical dilemmas, they may not strictly abide by the relevant code of conduct, but rather violate the rules for the benefit of the organization [32]. Based on the arguments presented above, this study proposes the following assumptions:

*Hypothesis 2a*: *Relational leadership negatively correlates with IEC*.
*Hypothesis 2b*: *Relational leadership negatively correlates with CEC*.
*Hypothesis 2c*: *Relational leadership positively correlates with REC*.

According to social construction theory, individuals interact with their environment to form their consciousness, emotions, and cognition, which when combined results in the construction of social culture and language, and the development of ways of thinking to match the situation and code of conduct. From this perspective, a “relationship” exists between people as a result of interpersonal interaction, as well as being a product of social construction. Thus, relational leadership is a process of social construction [13]. In this study, relational leadership denotes persistent concern for the career development of employees, for their empowerment, for opportunities in which they might offer suggestions, for planning good prospects for employees, and for treating them equally—all of which contributes to the creation of a friendly and caring ethical climate.

In organizations where IEC is prevalent, employees tend not to consider the potential consequences of their behavior. To pursue organizational goals such as productivity and efficiency, they often break rules and commit the unethical behavior [33]. In CEC, which focuses on collective interests, emotional communication between members and organizations is heightened through mutual help and respect. Employees feel care and support from leaders and the organization to such an extent that they may ignore immoral aspects of their behavior; this behavior is considered a kind of “sacrifice” for organizational interests [3,34,35]. In REC, employees follow clear behavioral norms: given ethical dilemmas they are guided and constrained by a broader range of ethical and legal standards. In addition, they tend neither to violate the rules for organizational purposes, nor help organizations through unethical means (that are either explicitly prohibited or at least not promoted).

Again, based on social construction theory employees form their consciousness, emotions, and cognition after being treated with fairness, vision, and empowerment by leadership. When employees perceive IEC, one would expect greater selfishness. To maximize self-interest, they could be expected to act in ways that further organizational interests and increase their own benefit; one would also assume greater ignorance of behavior that violates ethical standards. When employees perceive CEC, members of the organization help each other. To reciprocate leaders’ care and remain consistent with the values of the other members, one would expect greater ignorance of moral factors, and a tendency to unethical behavior that serves organizational interests. When employees perceive REC, owing to the existence of organization-defined codes of conduct, even in the face of ethical dilemmas they are more likely to abide by regulations, and less prone to putting organizational interests ahead of moral standards. Based on the arguments presented above, this study proposes the following assumptions:

*Hypothesis 3a*: *IEC mediates between relational leadership and UPB*.
*Hypothesis 3b*: *CEC mediates between relational leadership and UPB*.
*Hypothesis 3c*: *REC mediates between relational leadership and UPB*.

### 2.3 The moderating effect of moral identity

Moral identity reflects the individual’s recognition of moral qualities, such as fairness, caring for others, and loyalty, and is a crucial psychological mechanism for transforming moral cognition into moral behavior [36,37]. Currently, moral identity has gained prominence in the study of moral psychology [38]. Moral identity is extracted and activated in specific ethical situations, helping individuals to cope with ethics-based information, to make appropriate moral judgments, and to behave ethically. Research reveals that moral identity that exhibits a negative relationship with unethical behavior and negatively moderates the formation process of UPB [39,40,41]. According to self-consistency theory, when employees face an ethical dilemma, they compare their current self-moral image with the ideal moral image. To alleviate the psychological pressure created by the gap between reality and the ideal state, moral identity acts as a psychological adjustment mechanism—so that employees adjust their behavior to make it consistent with internal moral standards [42].

When employees perceive IEC, under a higher level of moral identity they perceive radical inconsistencies between the ethical climate and their moral level. Thus, employees adjust, emphasizing dedication and caring for others, and suppressing self-interested but unethical behaviors with respect to themselves. Conversely, given low moral identity amongst employees, the ethical climate is consistent with the prevailing moral level. In addition, employees are more concerned about their interests, and develop ostensibly unethical behavior to protect organizational interests [43,44,45,46]. When employees perceive CEC, under high moral identity they learn that the ethical climate corroborates with the moral level, thus emphasizing mutual help and caring for others. However, should employees exhibit unethical behavior whose goal was to protect organizational interests, the moral level would regulate such behavior so as to conform to that level, thereby restricting unethical behavior. Conversely, given low moral identity amongst employees, this factor would more readily be ignored in favor of organizational interests—and unethical behavior might follow [43,45,46]. Finally, when employees perceive REC, with heightened moral identity they realize that adherence to the organization’s code of conduct is a matter of safeguarding the organization’s interests. In such cases, employees would tend to comply with the organization’s regulations, avoiding the temptations of unethical behavior that might benefit the organization [45,46,47]. Conversely, when moral identity is low, employees are more likely to de-emphasize the organization’s code of conduct, to maintain a flexible, relaxed work environment, ignore the moral implications of their work, and, eventually, engage in unethical behavior. Based on the arguments presented above, this study proposes the following assumptions:

*Hypothesis 4a*: *Moral identity negatively moderates the relationship between IEC and UPB*. *For instance*, *the higher the level of employee moral identity*, *the weaker the effect of IEC on UPB*.
*Hypothesis 4b*: *Moral identity negatively moderates the relationship between CEC and UPB*. *For instance*, *the higher the level of employee moral identity*, *the weaker the effect of CEC on UPB*.
*Hypothesis 4c*: *Moral identity positively moderates the relationship between REC and UPB*. *For instance*, *the higher the level of employee moral identity*, *the stronger the effect of REC on UPB*.

**Fig 1:**
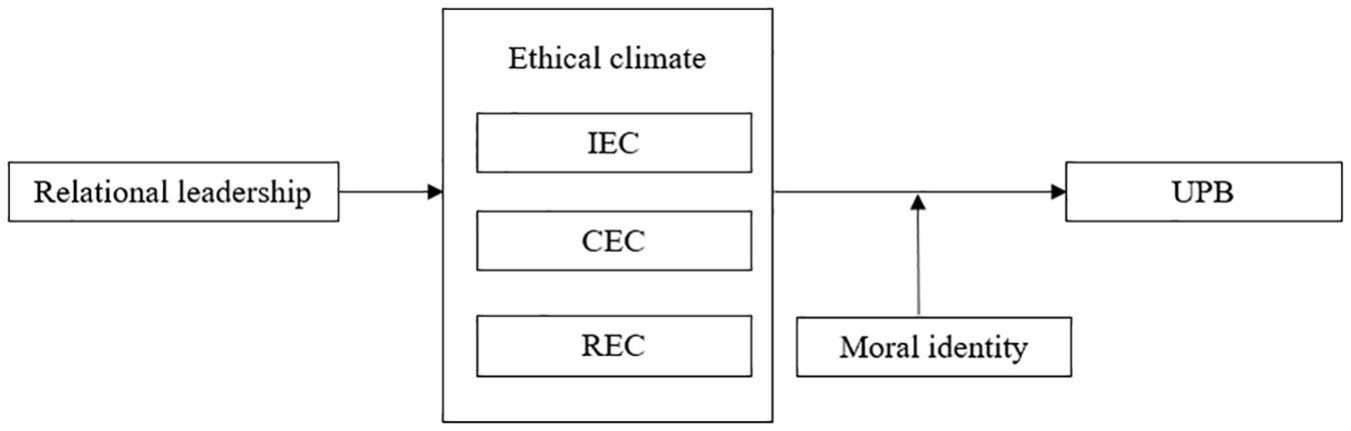
Theoretical model.

Fig 1 shows the theoretical model.

## Methods

### 3.1 Participants and procedures

In this study, we adopt two methods—email and field survey—and select as our research object the employees of tourism enterprises from four ASEAN countries. Of a total of 25 organizations, 11 were Thai companies, 7 Indonesian, 4 Singaporean, and 3 Malaysian. From each organization, we randomly selected 3–5 departments or other similar-sized work units, and then recruited 47 work units for research. All questionnaires were filled out anonymously by organization members; the first questionnaire survey was conducted with employees at the initial time-point (time-point 1). The time-point 1 questionnaire was given in a small envelope to employees based on the number, including perceived relational leadership, ethical climate, and demographic variables. At this stage, 500 people participated, from whom we collected 412 valid questionnaires. After 1 month (time-point 2), we conducted the second questionnaire survey with the same subjects. We distributed the remaining time-point 2 questionnaires, along with the envelope, to the corresponding employees based on the number. The questionnaires included moral identity and UPB. At this stage, we collected 376 questionnaires (including responses from 52 people who were away on business; for this subgroup, we distributed questionnaires via email). Notably, the questionnaire was sealed immediately upon completion, and the respondents were numbered after collection to ensure successful matching.

All respondents adhered to the principle of voluntary choice. Respondents whose answers were too short, who did not provide qualified answers, and who could not be contacted for a second or third survey, were eliminated from the study. Ultimately, we collected 325 valid questionnaires (questionnaire recovery efficiency, 63.00%). The gender breakdown was 51.99% male, 48.01% female. Further, participants aged ≤20 years accounted for 12.92%, 21–30 years for 27.69%, 31–40 years for 25.23%, 41–50 years for 18.15%, and ≥51 years for 16.01% of the respondents, respectively. In terms of educational level, junior college and below accounted for 61.23%, undergraduate for 32.62%, and graduate students for 6.15%. Regarding working years, ≤3 years accounted for 54.46%, 3–5 years for 24.62%, 5–10 years for 14.77%, and >10 years for 6.15%.

### 3.2 Measures

The initial questionnaire was written in English. We invited 3 English majors from Thailand, Indonesia, and Malaysia to translate the document into Thai, Indonesian, and Malay respectively. Subsequently we invited 3 human-resource scholars to check the scale. Of note, the 6 scholars mentioned above repeatedly compared notes, and ran numerous iterations of the traditional reverse translation method, until the English text bore close resemblance to the local version. In the study, we used the Likert-7 scale (1 = no match, while 7 = complete match; increasing from low to high). Given below are the existing maturity scales used in this study:

#### Relational leadership

The measurement of this variable is mainly based on the relational leadership scale developed by Carifio et al. [14], with a total of 25 items. The scale includes “leaders often care about our physical and mental health” and “leaders are good at adopting different opinions and opinions of employees.” In this study, Cronbach’s *α* coefficient of this scale was 0.824.

#### Ethical climate

A total of 13 items were used in the questionnaire prepared by the revised version proposed by Victor and Cullen [30]. Among them, six items were present in the IEC. For example, “our company wants employees to do anything for the benefit of the company regardless of the consequences”; Cronbach’s *α* coefficient was 0.853. The CEC utilized four items, such as “In our company, employees are related to each other”; Cronbach’s *α* coefficient was 0.761. The REC used three items. For example, “In our company, it is very important to comply with the rules and regulations”; Cronbach’s *α* coefficient was 0.747.

#### Moral identity

A total of five items, using a moral identity scale developed by Aquino et al. was used [36]; it includes “I especially want to have these qualities, such as fairness, friendliness, honesty and tolerance” and “I want to be a person with these qualities”; Cronbach’s *α* coefficient was 0.841.

#### Unethical pro-organizational behavior

In this study, the main reference was the UPB scale developed by Umphress et al. [3]. Absent items that were less appropriate to the ASEAN context. The scale has a total of five items; it mainly includes “If the company asks, I will exaggerate the quality of the company’s products or services” and “If it helps the company, I will distort the facts to maintain the image of the organization”; Cronbach’s *α* coefficient was 0.816.

#### Control variables

In this study, which was based on the study of employees’ UPB conducted by Umphress et al. and Matherne and Litchfield [3,48], we selected the following elements as control variables: gender of employees (dumb variables, 0 = female, and 1 = male), age (1 = 20 years; 2 = 21–30 years; 3 = 31–40 years; 4 = 41–50 years; and 5 = ≥51 years), education (1 = college and below; 2 = undergraduate; and 3 = graduate and above), working years (1 = ≤3 years; 2 = 3–5 years; 3 = foreign-funded enterprises; and 4 = joint ventures), and enterprise scale (1 = ≤50; 2 = 51–100 people; 3 = 5–10 years; and 4 = >10 years).

## Results

### 4.1 Confirmatory factor analysis

With the Mplus 7.4 analysis software, we tested discriminant validity by using the multifactor competition model. The six-factor model consisted of the following: relational leadership, IEC, CEC, REC, moral identity, and UPB; these were used as a benchmark and respectively compared with the other models. Table 1 shows that the fitting factors of the six-factor model are significantly better than those of the other factor models, which reached acceptable levels (*χ*^2^/df = 2.182, TLI = 0.978, CFI = 0.984, SRMR = 0.057, RMSEA = 0.057). Furthermore, these six main constructs exhibited good discriminant validity, and we conducted hypothesis testing in the following.

**Table 1:** Confirmatory factor analysis results.

Models	Factors	*χ*^2^/df	TLI	CFI	SRMR	RMSEA
Six-factor model	A, B, C, D, E, F	2.182	0.978	0.984	0.057	0.057
Five-factor model	A, B, C, D, E + F	2.634	0.813	0.826	0.063	0.071
Four-factor model	A, B + C + D, E, F	3.011	0.857	0.864	0.074	0.077
Three-factor model	A, B + C + D, E+ F	3.423	0.762	0.785	0.081	0.083
Two-factor model	A+B + C + D, E+ F	3.844	0.692	0.727	0.087	0.089
One-factor model	A + B + C + D + E+F	4.286	0.624	0.697	0.094	0.097

Note: A, B, C, D, E, F represent relational leadership, instrumental ethical climate, caring ethical climate, regular ethical climate, moral identity, and unethical pro-organizational behavior. “+” denotes the combination of two factors.

Meanwhile, relational leadership, IEC, CEC, REC, moral identity, and UPB were derived from employee reports, which could result in common method bias. To evade common method deviations because of homologous data, first, as far as process control was concerned, we indicated in the questionnaire that “this questionnaire is only used for academic research and not for commercial use,” and this study adopts a cross-point questionnaire collection method. Second, regarding method control, our study examined the common method bias by the confirmatory factor analysis based on the recommendations of Podsakoff et al. [49]. As shown in Table 1, the single factor fitting index (*χ*^2^/df = 4.286, TLI = 0.624, CFI = 0.697, SRMR = 0.094, RSMEA = 0.097) is not ideal. As the common method bias in the measurement of this study is not serious, the judgment of the common method bias did not significantly affect our findings.

### 4.2 Descriptive statistics and correlation analysis

Table 2 shows the mean, standard deviation, and correlation coefficient of the main variables. A significant negative correlation existed between relational leadership and IEC (*r* = –0.191, *p* < 0.05), which significantly positively correlated with the CEC (*r* = 0.214, *p* < 0.05) but did not significantly correlate with the REC (*r* = 0.071, *n*. *s*.), which significantly positively correlated with UPB (*r* = 0.214, *p* < 0.05). In addition, the IEC (*r* = 0.274, *p* < 0.001) and CEC (*r* = 0.302, *p* < 0.001) significantly positively correlated with employees’ UPB, and the REC significantly negatively correlated with UPB (*r* = –0.218, *p* < 0.05). The above relevant results provide preliminary support for our research hypothesis.

**Table 2:** Mean, standard deviation, and correlation coefficient matrix of the main variables.

	1	2	3	4	5	6
1 Relational leadership	1					
2 IEC	–0.191*	1				
3 CEC	0.214*	0.487***	1			
4 REC	0.071	0.524***	0.511***	1		
5 Moral identity	–0.152*	–0.351***	–0.218*	–0.241**	1	
6 UPB	–0.214*	0.274***	0.302***	–0.218*	0.212*	1
Mean	4.231	3.842	4.375	4.197	3.214	4.571
SD	1.114	1.342	1.023	1.354	1.117	1.194

Note: *n* = 325,

*** *p* < 0.001,

** *p* < 0.01,

*p < 0.05;

Two-tailed test.

### 4.3 Hypothesis testing

#### (1) Testing the mediation effect

Before regression analysis, a multicollinearity test was performed on all variables, which revealed that VIF values of all regression models were <2, and tolerance was >0.100, suggesting no serious multicollinearity. We used STATA 14.0 for the hierarchical regression analysis; Table 3 presents the results. Regarding the assumption of the relationship between relational leadership and ethical climate, relationship leadership significantly negatively affected the IEC (M2, *b* = – 0.324, *p* < 0.01), thereby supporting H2a. Relationship leadership significantly positively affected CEC (M4, *b* = 0.253, *p* < 0.01), thereby supporting H2b. Furthermore, relational leadership exerted no significant impact on REC (M6, *b* = −0.013, *n*. *s*.), thereby lending no support to H2c.

**Table 3:** Hierarchical regression analysis results of ethical climate.

	IEC		CEC		REC	
	M1	M2	M3	M4	M5	M6
**Control variables**						
Gender	–0.157*	0.129	–0.035	0.025	0.052*	0.014
Age	–0.073	–0.058	0.041	0.023	0.034	0.042
Education	0.052	0.038	–0.068*	–0.046	–0.027	–0.090
Working years	–0.134*	0.054	–0.034	0.038	0.024	0.014
**Independent**						
Relational leadership		–0.324**		0.253**		–0.013
R^2^	0.021	0.083	0.028	0.097	0.032	0.055
ΔR^2^		0.062**		0.076***		0.023

注: *n* = 325,

*** *p* < 0.001,

** *p* < 0.01,

* *p* < 0.05.

Regarding the hypothesis of the ethical climate mediating, Table 4 shows that relational leadership, IEC, and CEC, significantly positively affected employees’ UPB (M9, *b* = 0.267, *p* < 0.001; *b* = 0.254, *p* < 0.001; *b* = 0.262, *p* < 0.001), and REC significantly negatively affected UPB (M9, *b* = –0.214, *p* < 0.001). Table 4 shows that relational leadership positively correlates with employees’ UPB (M8, *b* = 0.383, *p* < 0.001), thereby supporting H1. To further validate the mediation effect of the ethical climate, we used Mplus 7.4 to test the Bootstrap mediation effect (Table 5). The bias-corrected 95% confidence interval for relational leadership through IEC and CEC to employees’ UPB was [0.112, 0.283] and [0.134, 0.291], excluding 0, suggesting that IEC and CEC existed for the mediation effect of relational leadership and employees’ UPB—thereby supporting H3a and H3b. The bias-corrected 95% confidence interval for relational leadership through REC to employee UPB was [–0.121, 0.218], including 0, demonstrating that the mediation effect of REC on the relationship between the relational leadership and employee UPB does not exist, thereby offering no support for H3c.

**Table 4:** Hierarchical regression analysis results of UPB.

	UPB						
	M7	M8	M9	M10	M11	M12	M13
**Control variables**							
Gender	–0.011	–0.018	–0.021	–0.024	–0.027	0.032	–0.036
Age	–0.026	–0.036	–0.049*	–0.051*	–0.037	0.042	–0.036
Education	0.019	–0.013	–0.028	–0.022	–0.027	0.026	0.039
Working years	0.031	0.024	–0.018	0.025	0.022	0.013	0.017
**Independent**							
Relational leadership		0.383***	0.267***	0.245***	0.228***	0.232***	0.226***
**Mediator**							
IEC			0.254***	0.235***	0.217***	0.226***	0.211***
CEC			0.262***	0.241***	0.233***	0.238***	0.224***
REC			−0.214**	−0.198**	−0.193**	−0.196**	−0.187**
**Moderator**							
Moral identity				0.218**	0.212**	0.214**	0.206**
**Interaction term**							
IEC × Moral identity					−0.084*		
CEC × Moral identity						−0.076*	
REC × Moral identity							0.082*
R^2^	0.034	0.179	0.256	0.279	0.294	0.306	0.286
ΔR^2^		0.145***	0.077***	0.023***	0.015***	0.027***	0.007*

Note *n* = 325,

*** *p* < 0.001,

** *p* < 0.01,

* *p* < 0.05.

**Table 5:** Mediation effect test.

Variables	Bootstrapping			
	Bia-Corrected 95% CI		Percentile 95% CI	
	Lower	Upper	Lower	Upper
Indirect effect				
Relational leadership → IEC →UPB	0.112	0.283	0.109	0.279
Direct effect				
Relational leadership → IEC →UPB	0.321	0.580	0.325	0.583
Indirect effect				
Relational leadership → CEC →UPB	0.134	0.291	0.131	0.287
Direct effect				
Relational leadership → CEC →UPB	0.311	0.571	0.314	0.576
Indirect effect				
Relational leadership → REC →UPB	−0.121	0.218	-0.123	0.220
Direct effect				
Relational leadership → REC →UPB	0.105	0.304	0.109	0.307

Note: n = 325, Bootstrapping random sampling 2000 times.

#### (2) Testing the moderator effect

**Fig 2:**
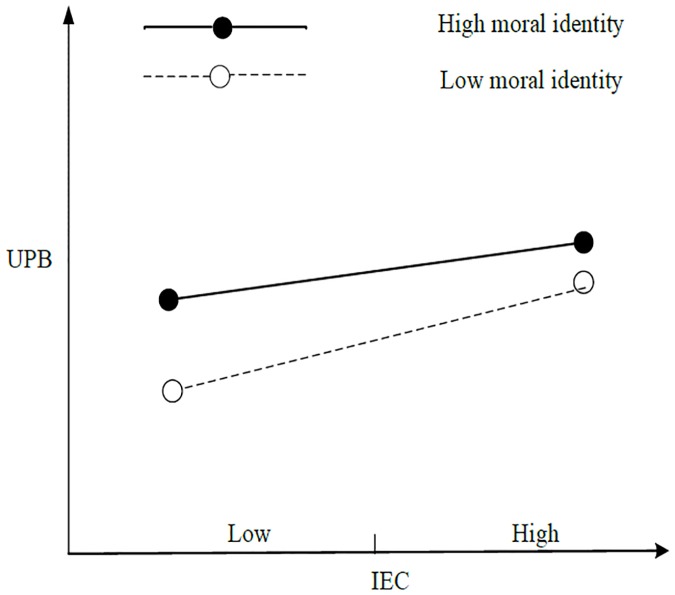
Simple slope test for IEC.

**Fig 3:**
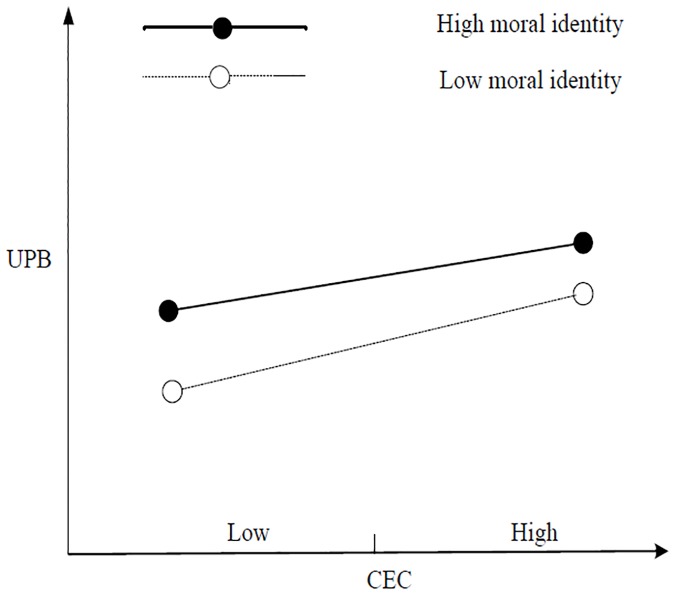
Simple slope test for CEC.

**Fig 4:**
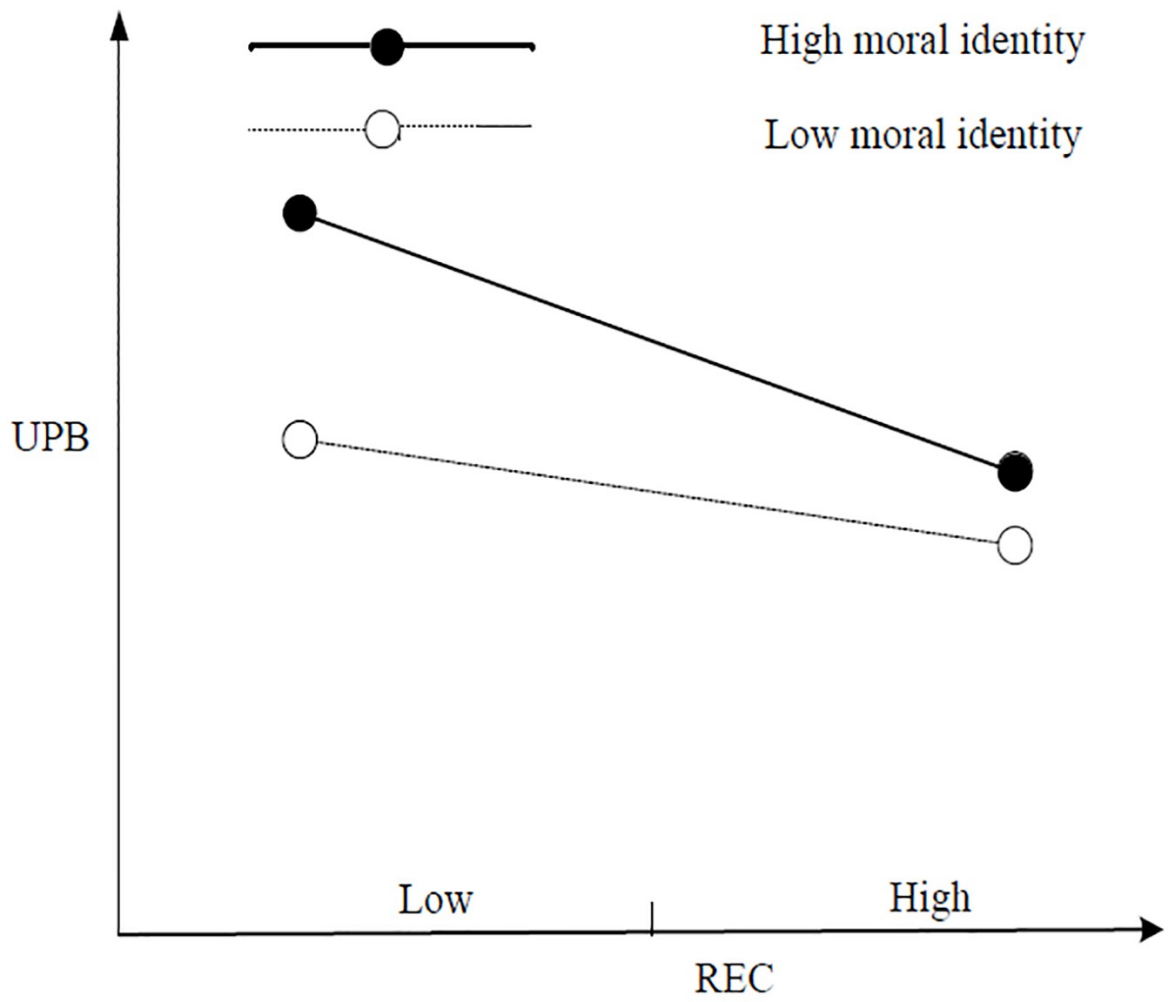
Simple slope test for REC.

First, Table 4 shows that the interaction items of IEC and moral identity, as well as those of CEC and moral identity, exerted a significant negative impact on UPB (M11, *b* = –0.084, *p* < 0.05; M12, *b* = –0.076, *p* < 0.05), and the interaction term between REC and moral identity exerted a significant positive impact on UPB (M13, *b* = 0.082, *p* < 0.05). The results of the simple slope test revealed that under high moral identity (mean + 1 standard deviation), IEC exerted no significant impact on UPB (*b* = 0.042, *n*. *s*.). Under low moral identity (mean– 1 standard deviation), IEC exerted a significant positive impact on UPB (*b* = 0.226, *p* < 0.001), thereby supporting H4a. Fig 2 shows a simple slope test. Under high moral identity (mean + 1 standard deviation), CEC exhibited no significant impact on UPB (*b* = 0.076, *n*. *s*.); under low moral identity (mean– 1 standard deviation), CEC exerted a significant positive impact on UPB (*b* = 0.234, *p* < 0.001), thereby supporting H4b. Fig 3 shows the simple-effect test. Under high moral identity (mean + 1 standard deviation), REC exhibited a significant negative impact on UPB (*b* = –0.281, *p* < 0.001); under low moral identity (mean– 1 standard deviation), REC exerted no significant impact on UPB (*b* = –0.081, *n*. *s*.), thereby supporting H4c. Fig 4 shows a simple slope test.

## Discussion

Based on the theory of social construction and self-consistency, this study demonstrates the mechanism of relational leadership’s role in employees’ UPB from the perspective of moral identity and ethical climate. The findings reveal that relational leadership negatively correlates with IEC, positively correlates with CEC, and exerts no significant impact on REC. In addition, IEC and CEC mediated the relationship between relational leadership and employees’ UPB. Moral identity negatively moderates the relationship between IEC and employee UPB and CEC. When employees’ moral identity is higher, the IEC and CEC exert less impact on the promotion of employee UPB. Moral identity positively moderates the relationship between REC and employee UPB. When employees’ moral identity is higher, the restraining effect of the REC on the employee UPB is weaker. In this study, two hypotheses received no support: (i) the relationship between relational leadership and REC and (ii) the mediating role of REC. One probable reason is that relational leadership might have paid more attention to their self-cultivation and self-discipline, and so strictly abide by the organization’s rules and regulations. Based on social learning theory, individuals perceive the way things are handled through observation and example, imitating the behavior of the model meanwhile. In organizations, the leader is the object of employee imitation, and the leader’s code of conduct is the norm for employee learning. Thus, the relationship leader is more regular, and the employee imitates and strictly abides by laws, regulations, and ethics. Moreover, the negative impact of relational leadership on REC is weakened, resulting in a nonsignificant relationship between the two. In addition, because of the nonsignificant relationship between the first-stage relational leadership and REC, even if the hypothesis supports the relationship between REC and UPB, the mediating role of REC has not been realized.

### 5.1 Theoretical implications

First, there is a need to focus on UPB (which is often overlooked by leaders), and to expand its incentive system. As the proverb “The road to hell is paved with good intentions” suggests, this study found that positive leadership style does not necessarily yield positive effects. On one hand, previous studies primarily focused on transformational leadership, ethical leadership, and other leadership styles [7,8,9]. As a caring leadership style, relational leadership is common in modern organizations. However, no study to date has explored the relationship between relational leadership and UPB; hence, our study attempted to supplement it. On the other hand, this paper elucidates the role of relational leadership in promoting employee UPB, and provides reference and inspiration for further research. In addition, extant studies have overemphasized the management philosophy of “humanism.” Organizations not only keep silent or neglect employee UPB; they sometimes actually encourage it [50]. While ostensibly beneficial to the organizations in question, in the long run this endangers the development of the entire organization, and possibly even the whole industry. By discussing the formation mechanism of UPB in ASEAN organizations—and from the perspective of relational leadership—our study addresses the promotion effect of relational leadership on employee UPB, and provides theoretical reference for subsequent research on relational leadership and employee UPB. This not only augments scholarly and managerial understanding of the harms of UPB; it also expands the antecedent variables affecting UPB [6,51].

Second, a path between relational leadership and UPB was added. Employees form their consciousness and cognition under the influence of different environments or leadership style; such construction often forms an active consciousness and cognition in an active leadership style. Prior studies often address the impact of leadership style on UPB from the perspective of employees such as organizational identity [7,8,9]. As an organizational environment, ethical climate exerts a subtle influence on employee behavior; however, these studies remain limited [52,53]. We find that IEC and CEC realize the transfer of the role of relational leadership to employee UPB. While relational leadership is a style that accentuates caring and embracing employees, it increases employee UPB. When such leadership demonstrates care for an employee, then if that employee perceives IEC, he/she might easily be motivated towards self-interest, which is both legitimate and reasonable. Notably, taking any means to protect the interests of an organization is natural enough. (Moreover, the leader is attuned to an employee’s concern, tolerance, and authorization; the individual then tends to develop some ostensibly unethical behavior to organize the interest [8,22]. When employees perceive CEC, they feel valued by leaders and organizations; leaders care for them. As an ethical climate emphasizes mutual help and mutual trust, it is easy to conceal the individual’s capacity for moral judgment, which in turn may lead an individual to ignore ethics in favor of collective benefit, and thus regard UPB as a kind of spirit of sacrifice (for self and for organization this demonstrates that although the CEC is a positive ethical climate, there is an attendant risk of inducing individuals to do “bad things.” Furthermore, discussion on the mediating effect of ethical climate not only complements and improves social construction theory, but also reveals and begins to enrich a new path between relational leadership and UPB. Then too, it helps clarify the mechanism of action between relational leaders and employee UPB.

Third, we have identified a new boundary condition between ethical climate and UPB. Previously, research on employee UPB mainly relied on the relationship between cultural organization and organizational relations such as leadership organization and collectivism [54,55]. Unethical behavior is essentially an unethical act; therefore, it is particularly critical to look at the moral character of employees. Hence, this study reveals that when employee moral identity is low, IEC and CEC increase the UPB. In addition, employees learn that IEC is more consistent with their ethical standards; they then tend to exhibit unethical behavior to satisfy their self-interest, regardless of moral constraint. The CEC underlines mutual help and an atmosphere of mutual concern. If the level of ethics is low, employees ignore this unethical behavior for organizational purposes and thus tend to develop UPB. When the employee moral identity is high, REC exhibits a stronger inhibitory effect on UPB. If employees encounter UPB, they make genuine attempts to remain consistent with their high moral standards, and so comply with relevant laws, regulations, and rules of social ethics, all while avoiding unethical behavior for the benefit of the organization. Moreover, our findings might not only enhance the understanding of the ethical climate and boundary conditions between UPB, but also expand and supplement the research field of moral identity.

### 5.2 Managerial implications

The conclusions of this study have crucial practical guiding significance for organizational management practice.

First, leaders should avoid giving employees too much care and authorization Leaders should be aware of the negative impact that over-care and empowerment exerts on employees. Leaders can learn the degree of leadership care, authorization, and other employee behavior through anonymous employee surveys, enabling them to determine whether relationship leaders are acting excessively and so to prevent the generation and expansion of negative impacts. In addition, leaders need to respect employees, focus on all aspects of employee needs, and actively mobilize employee initiative with respect to participation in decision-making processes. Besides, leaders could be appropriately authorized to employees, create a fair, caring, and rule-oriented ethical atmosphere, and establish a firm and reasonable reward-and-punishment mechanism. It is imperative to take advantage of relational leadership, and to avoid the negative impacts of excessive care for employees.

Second, organizations should enhance the level of employee moral identity. In the ever-evolving market competition environment, employees often face changes and uncertainties at work. This is especially true for tourism-oriented enterprises, where there is great diversity in customer quality and in culture, and where most clients are short-term customers, a fact that poses even greater challenges for organizations that operate in the industry. Accordingly, the level of moral identity in these organizations is particularly crucial. It is incumbent on leaders to focus on the moral level, to lead by example, to act as moral role models, to ensure high moral standards, and to recognize their employees. Such an approach might better avoid or decrease the impact of employee UPB on the organization, and more generally help it to develop healthily and sustainably.

Third, organizations should ensure positive ethical climates. They ought to suppress and eliminate internal selfishness and self-interest, and strive to prevent the creation and spread of IEC. In turn, they should develop and implement employee ethical-behavior guidelines. Further, organizations should embody ethical corporate cultures, create positive and regular ethical climates, and establish sound moral training mechanisms, including provisions for reward, punishment, and protection. Ideally, these steps would enable employees to comply with organizational ethical rules, and improve their own ethical standards. Moreover, such measures might inhibit the generation of UPB, thereby better assisting in the ongoing healthy development of the organization. While striving to create CEC, organizations should also focus on ethical attributes, and persuade employees to reward organizations and help others correctly and ethically.

### 5.3 Limitations and future research

The study has some limitations, and room for future improvement.

First, although we collected our data in a time-sharing manner, all questionnaires were reported by employees. Whether the leader’s evaluation corroborates the actual situation needs to be reported by the leader him/herself. Thus, in any follow-up study, various data collection channels should be added, and employees, leaders, and peers should be selected as subjects to assess and maintain, to the extent possible, the authenticity and validity of the data. Second, the theory and scale used in this study are derived from Western literature. Applicability to the ASEAN region warrants further testing. In the same vein, future research should focus on the development and use of ASEAN countries’ situational and localized measurement tools. Third, our study is limited to an investigation of the impact of ethical climate and moral identity (at the individual perception level) on the formation process of employee UPB. In reality, other variables at the individual level, leadership style at the leadership level, and organizational ethics at the organizational level could also affect UPB, necessitating the expansion of research in this area. Currently, cross-level research has become a research trend. The comprehensive cross-influence of the organizational level and leadership to individual UPB helps to more fully elucidate the formation mechanism of UPB; this also constitutes a promising future research direction. Fourth, in recent years, the moderated mediation model has become increasingly popular among scholars, and it is helpful for us to better understand the theoretical model in this paper by subjecting it to examination. Unfortunately, our data do not support the existence of a moderated mediation effect. Possible reasons are as follows: (1) Our data are from four countries, whose cultural differences may lead to the strength of moral identity, which can only support hypothesis 4a-4c statistically. (2) Relational leadership influence ethical climate, ethical climate influence UPB of these two paths is not completely consistent (some are positive, some are negative), which is likely to lead to cancel each other out by the moderated mediation effect, in addition, given by the moderated mediation effect of more statistically significance of the above, therefore, we don’t have to put this hypothesis is put forward in this paper. However, whether the above reasons or the moderated mediation effect does not exist needs to be further discussed in the future.

## Conclusions

Based on social construction theory and self-consistency theory, this study investigates how relational leadership affects the ethical climate, which in turn affects employee UPB; it further assesses the boundary conditions of the relationship from the perspective of moral identity. Our findings reveal that relational leadership positively affects employee UPB, and the process is passed through IEC and CEC. Negative moral identity moderates the relationship between and amongst IEC, CEC, and UPB, while moral identity positively moderates the relationship between REC and UPB.

On the one hand, this study starts with relational leadership and establishes it as a critical factor in promoting employee UPB. In addition, the process is passed through IEC and CEC, which uncovers the black box of relational leadership influence on employee UPB. On the other hand, we focus on the boundary conditions of the relationship, finding that when employee moral identity is high, employee concerns and authorization from leaders exert different impacts on UPB because they perceive different ethical climates. Furthermore, the research results provide theoretical reference and empirical support for how to correctly view relational leadership, for guiding employees to create a positive ethical climate under relational leadership, and for inhibiting employee UPB.

## Supporting information

S1 File Data (Thailand + Singapore + Malaysia). (XLSX)
